# Effects of Folate and Fructose Intakes on Renal Cytokines and Fibrosis in an Adenine-Induced Mouse Model of Chronic Kidney Disease

**DOI:** 10.3390/ijms27010499

**Published:** 2026-01-03

**Authors:** Ting-Yu Chen, Ya-Ching Chiu, Bi-Fong Lin

**Affiliations:** Department of Biochemical Science and Technology, College of Life Science, National Taiwan University, Taipei 10617, Taiwan; r11b22026@ntu.edu.tw (T.-Y.C.); r11b22006@ntu.edu.tw (Y.-C.C.)

**Keywords:** folate, high-fructose diet, chronic kidney disease, fibrosis, adenine-induced nephropathy

## Abstract

Dietary pattern characterized by low intake of vegetables and fruits and high consumption of fat, soft drink and desserts are associated with an increased risk of chronic diseases. To investigate the effects of folate status and fructose intake on adenine-induced chronic kidney disease (CKD), seven-week-old C57BL/6 mice were divided into six groups and fed either a control diet (Ctrl), a 26% (*w*/*w*) high-fructose diet (Hfru), Ctrl plus 0.15% adenine (Ctrl+ade), Hfru+ade, Hfru with folate deficiency plus adenine (Hfru−f+ade), or Hfru with tenfold folate supplementation plus adenine (Hfru+f10+ade). After 10 weeks on the assigned diets, adenine was administrated to the +ade groups for 7 weeks. The results showed that all adenine-treated mice exhibited increased fasting blood glucose, urinary glucose, and elevated renal expression of collagen 1a1 (*Col1a1*), fibronectin (*Fn1*), and smooth muscle α-actin (*Acta2*). Compared with Ctrl mice, Hfru-fed mice showed significantly higher serum creatinine, increased urinary protein, and reduced creatinine clearance. Adenine induced kidney injury in all +ade groups, with the most severe damage observed in Hfru−f+ade mice, as indicated by elevated blood urine nitrogen (BUN), urinary protein, neutrophil gelatinase-associated lipocalin (NGAL), and renal fibrosis. In contrast, Hfru+f10+ade mice showed the lowest levels of these renal injury markers. The Hfru+ade diets increased renal *Hif1α* and *iNos* gene expression, which was further exacerbated by folate deficiency. Secretion of the anti-inflammatory cytokine interleukin (IL-10) by splenocytes was significantly reduced under folate-deficient conditions. Renal IL-10 levels were suppressed in all +ade groups but were significantly increased by folate supplementation. Renal IL-10 levels were negatively correlated with the inflammatory chemokine monocyte chemoattractant protein (MCP-1) and transforming growth factor (TGF)-β, whereas renal MCP-1 levels showed positive correlations with TGF-β and IL-6. Overall, these findings suggest that high fructose consumption in the absence of adequate folate intake may be of concern for CKD progression.

## 1. Introduction

The prevalence of chronic kidney disease (CKD) is increasing in aging societies, as aging is associated with a gradual decline in physical and mental function at the cellular, molecular, and systemic levels. Although the kidney does not possess the same regenerative capacity as the liver, it remains a limited ability to repair localized injury. Mild renal damage may be partially reversible; however, severe injury can result in permanent loss of function. Therefore, it is important to investigate whether imbalanced dietary patterns adversely affect kidney health in industrialized and aging populations. In modern lifestyles, diets high in fat, sugar, fructose syrup, desserts, and fast food are increasingly common are often accompanied by reduced vegetable intake [1]. However, because the kidney can recover from mild injury, it may take considerable time for unhealthy dietary patterns to result in overt kidney failure. Our previous studies showed that C57BL/6 mice did not develop significant renal damage until after 12-months of high-fat, high-fructose feeding, although transient proteinuria was observed in some mice and subsequently resolved during the experimental period [2].

Adenine-induced nephropathy has recently been used as a model to study CKD because it mimics several key features of human disease, including inflammation, fibrosis, and oxidative stress [3]. The C57BL/6 mouse strain is known to be relatively resistant to fibrosis compared with the other strains [4,5]. Therefore, we aimed to investigate whether adenine supplementation could accelerate CKD development in mice fed a high-fat, high-fructose diet. Unlike acute kidney injury, CKD is characterized by a gradual decline in kidney function, including reduced ability to filter waste products and excess fluid from the blood. Persistent inflammation is a major driver of CKD progression, leading to progressive tissue damage over time. In CKD, inflammatory molecules such as cytokines remain chronically elevated, contributing to fibrosis, oxidative stress, and further deterioration of kidney function.

Supporting the hypothesis that dietary factors play a role in kidney health, our previous study demonstrated that gamma-aminobutyric acid (GABA) and GABA-enriched rice suppressed inflammatory responses—including serum interleukin (IL)-6, renal monocyte chemoattractant protein (MCP)-1, transforming growth factor (TGF)-β, and IL-17A levels—and reduced macrophages infiltration in the kidney, thereby alleviating renal injury in a von Hippel-Lindau gene knockout CKD-prone mice model [6]. Another cytokine, IL-10, is a key anti-inflammatory mediator that protects against kidney injury by suppressing inflammation and fibrosis [7]. A recent review reported that IL-10-based immunotherapy reduced the expression of TNF-α, IL-6 and IL-1β, promoted M2 macrophage polarization, and decreased tubular injury scores and long-term kidney fibrosis in animal models of renal ischemia-reperfusion injury, highlighting the therapeutic potential of IL-10 in clinical applications [8].

TGF-β is a central mediator of fibrosis in CKD, exerting a dual role by suppressing inflammation while promoting fibrotic progression. In addition to TGF-β, other key indicators of renal fibrosis include *Col1a1* expression, which encodes the alpha-1 chain of type 1 collagen; *Fn1*, which encodes fibronectin 1; and *Acta2*, which encodes smooth muscle α-actin, α-SMA. These markers are widely recognized as indicators of fibrotic progression in CKD.

In CKD, hypoxia is both a consequence and a contributing factor to disease progression. Renal injury reduces blood flow, leading to chronic oxygen deficiency, which in turn promotes inflammation and fibrosis. Hypoxia-inducible factor (HIF) is activated under these conditions and plays a protective role in the hypoxic response [9]. Specially, HIF-1 can upregulate inducible nitric oxide synthase (*iNos*) expression, increasing nitric oxide synthesis to limit ischemic damage, which contributes to the elevated renal *iNos* expression commonly observed in CKD [10].

In modern life, folate insufficiency has become increasingly prevalent due to a combination of dietary habits and lifestyle factors. Although folate is naturally present in leafy greens, legumes, and fortified grains, many people rely heavily on processed or convenience foods that relatively low natural vitamins. Busy lifestyles, restrictive diets, and dietary trends that limit grain consumption may further reduce folate intake. Additionally, alcohol consumption, certain medications, and chronic stress can impair folate absorption or utilization, particularly in older adults. As a result, low folate status remains a public health concern even in food-abundant societies, with potential consequences for energy metabolism, DNA synthesis, and overall health.

Previous studies have shown that folate deficiency impairs dendritic cell function—the most abundant resident immune cell in the normal kidney—and subsequently alters T-helper cell differentiation [11]. Folate deficiency has also been shown to significantly increase MCP-1 and IL-6 production in 3T3-L1 cells, including both in preadipocytes and adipocytes [12]. To model a modern dietary pattern characterized by high intake of processed foods rich in fat and sugar or fructose syrup and reduced vegetable consumption, our previous study demonstrated that folate deficiency elevated renal IL-6, MCP-1, and TGF-β levels while decreasing IL-10 in mice fed either a normal diet or a high-fat (45% Kcal from fat; 23.6% *w*/*w*), high-fructose (31.6% *w*/*w*) diet for 12 months [2]. Collectively, these findings indicate that folate status is crucial for maintaining immune homeostasis and limiting inflammatory responses.

Few studies have examined the combined effects of folate status and fructose intake on CKD. The present study aimed to investigate the impact of folate status and high-fructose intake, in the context of a reduced-fat diet, on CKD development. Accordingly, C57BL/6 mice were fed a medium-fat (12% *w*/*w*), high-fructose (31.6% *w*/*w*) diet with either folate deficiency or folate supplementation at ten times the level of the control diet. This experimental design allowed us to determine whether dietary folate modulates pro- and anti-inflammatory cytokines (TNF-α, IL-6, MCP-1, and IL-10), as well as the expression of *Hif1α* and *iNos*, in mice fed a high-fructose diet. Renal TGF-β levels and fibrosis-related gene expression (*Col1a1*, *Fn1*, and *Acta2*) were also assessed. Overall, this study evaluated the effects of folate status and fructose intake on kidney function, inflammation, and fibrosis in an adenine-induced murine model of CKD.

## 2. Results

### 2.1. Animals

**Figure 1:**
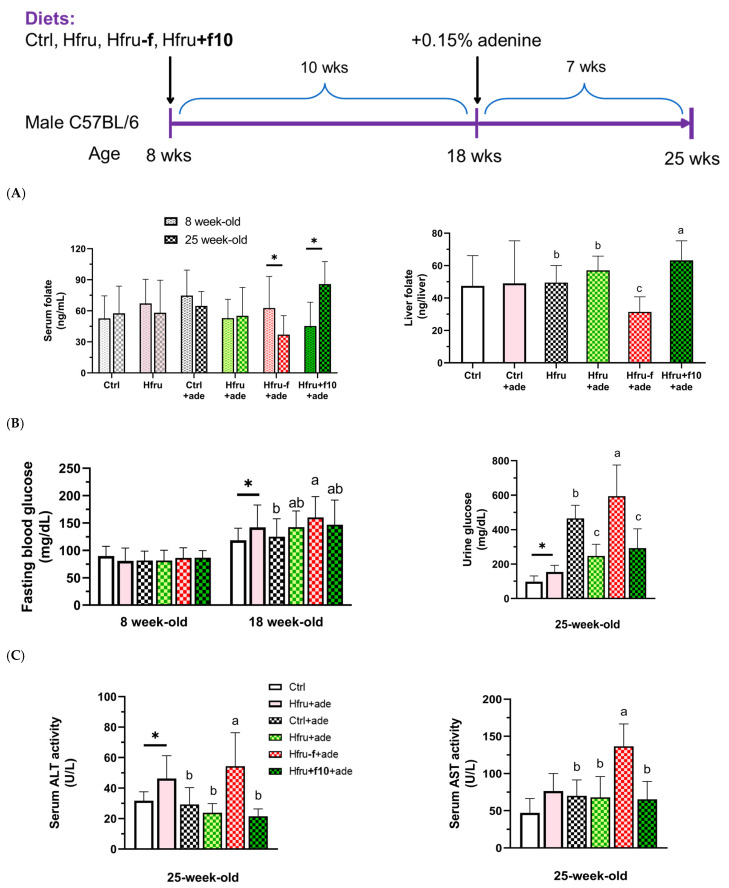
Experimental design of the study and metabolic parameters in an adenine-induced murine model of chronic kidney disease (CKD). (**A**) serum and hepatic folate levels in mice before (8-week-old) and after (25-week-old) the experiment diets. (**B**) fasting blood glucose and urinary glucose levels. (**C**) serum alanine aminotransferase (ALT) and aspartate aminotransferase (AST) activities. Data are presented as mean ± SD (n = 12 per group). * *p* < 0.05 versus the Ctrl group. Different letters indicate significant differences among adenine-induced groups (*p* < 0.05; (**A**) one-way ANOVA with Duncan’s post hoc test, (**B**,**C**) Kruskal–Wallis tests followed by Bonferroni-corrected Mann–Whitney U post hoc tests).

There were no significant differences in initial body weight among the control and experimental groups. As shown in the experimental design (Figure 1), after 10 weeks of dietary intervention, mice in the Hfru group had significantly higher body weight than those in the control group (37.2 ± 3.5 g vs. 32.5 ± 3.2 g). The Hfru−f+ade mice tended to have lower body weight than the Hfru+f10+ade mice, although this difference did not reach statistical significance (33.6 ± 4.1 g vs. 35.4 ± 3.2 g). Seven weeks of adenine administration resulted in a slight reduction in body weight, likely due to decreased food intake.

Folate status was assessed by measuring serum and hepatic folate concentrations (Figure 1A). After 17 weeks of folate-deficient or folate-supplemented feeding, Hfru−f+ade mice had significantly lower, whereas Hfru+f10+ade mice showed significantly higher, serum and hepatic folate levels. Significantly elevated fasting blood glucose and urinary glucose levels were observed in the Hfru groups, with the highest levels detected in the Hfru−f+ade mice compared with the control group (Figure 1B).

Serum alanine aminotransferase (ALT) and aspartate aminotransferase (AST) activities were measured as indicators of hepatic and systemic tissue injury (Figure 1C). ALT activity was significantly elevated in the Hfru and the Hfru−f+ade groups, but not in the other ade groups, compared with the Ctrl group. These findings indicate that folate deficiency exacerbates metabolic dysregulation induced by a high-fructose diet.

### 2.2. Kidney Function

**Figure 2:**
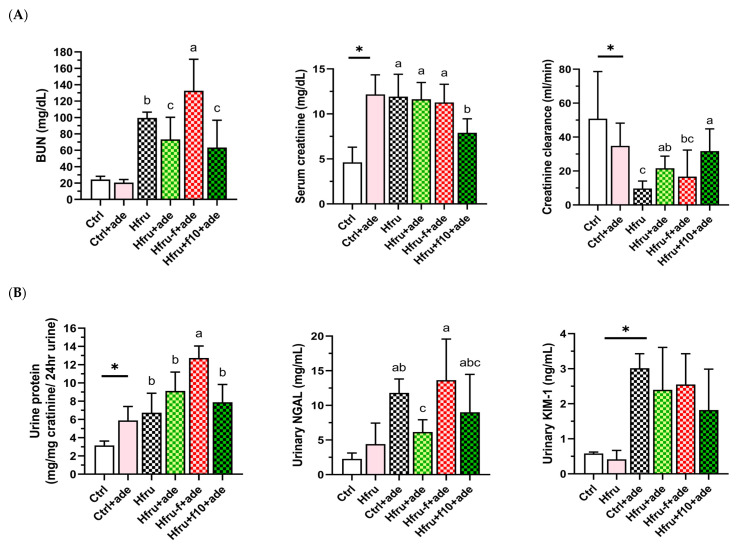
Kidney function parameters in an adenine-induced murine model of CKD under dietary folate and fructose conditions. (**A**) blood urea nitrogen (BUN), serum creatinine, and creatinine clearance. (**B**) twenty-four-hour urinary total protein, neutrophil gelatinase–associated lipocalin (NGAL), and kidney injury molecule-1 (Kim-1). Values are mean ± SD (n = 12 per group). * *p* < 0.05 versus the Ctrl group. Different letters indicate the significant differences among adenine-induced groups. (*p* < 0.05; (**A**) one-way ANOVA with Duncan’s post-hoc test, (**B**) Kruskal–Wallis tests followed by Bonferroni-corrected Mann–Whitney U post hoc tests).

After 7 weeks of adenine induction, serum markers of renal injury, including blood urea nitrogen (BUN) and creatinine, were significantly elevated, and creatinine clearance was significantly reduced in all adenine-treated groups (Figure 2A). Urinary markers of kidney injury, including total urine protein, urinary kidney injury molecule-1 (KIM-1) and neutrophil gelatinase-associated lipocalin (NGAL), were also significantly increased (Figure 2B).

Compared with Ctrl mice, Hfru mice exhibited significantly higher serum creatinine levels, increased urinary protein, and reduced creatinine clearance. Among all groups, Hfru−f+ade mice showed the most severe renal injury, with significantly elevated BUN, urinary protein, and NGAL levels. In contrast, Hfru+f10+ade mice exhibited the lowest BUN and creatinine levels and the highest creatinine clearance among the adenine-treated groups. These data indicate that high-fructose feeding independently impaired renal function, as evidenced by proteinuria, while folate supplementation attenuated and folate deficiency exacerbated adenine-induced renal injury.

### 2.3. Renal Fibrosis

**Figure 3:**
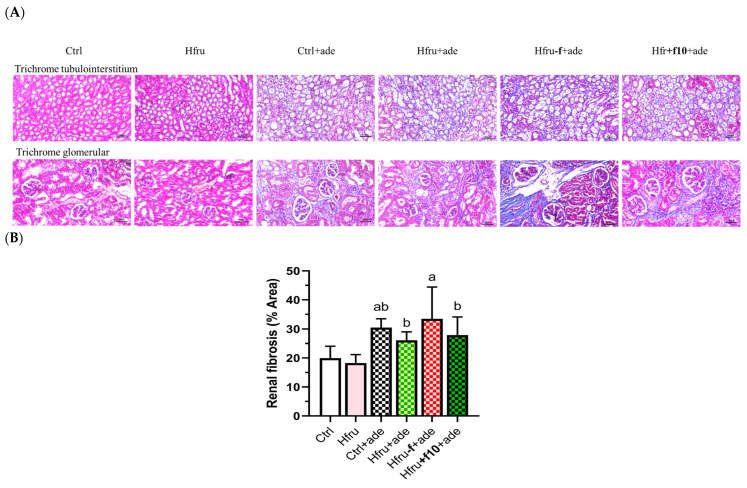
Histological assessment of renal fibrosis in adenine-induced CKD mice under different dietary folate conditions. (**A**) representative images of Masson’s trichrome-stained kidney sections (tubulointerstitium, 200×; upper panel; cortex, 200×, lower panel), scale bar = 50 μm. (**B**) quantification of renal fibrosis area from trichrome-stained sections using image analysis software. Different letters indicate significant differences among adenine-induced groups (*p* < 0.05; one-way ANOVA with Duncan’s post-hoc test).

Representative Masson’s trichrome-stained kidney sections are shown in Figure 3A. Trichrome staining of the tubulointerstitium was used to evaluate scarring or fibrosis by assessing collagen deposition, while glomerular staining also reflected fibrotic changes. Hfru−f+ade mice exhibited the highest percentage of fibrotic area (Figure 3B).

**Figure 4:**
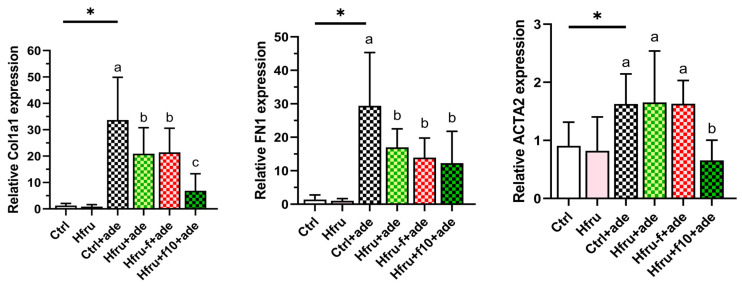
Expression of renal fibrosis-related genes in an adenine-induced murine CKD model under dietary folate conditions. Relative gene expression levels were normalized to *GAPDH*. Values are mean ± SD (n = 12 per group). * *p* < 0.05 versus the Ctrl group. Different letters indicate significant differences among adenine-induced groups (*p* < 0.05; one-way ANOVA with Duncan’s post-hoc test).

Fibrosis-related gene expression in renal tissue was also evaluated. As shown in Figure 4, Ctrl+ade mice exhibited the highest expression levels of *Col1a1*, *Fn1*, and *Acta2*. Because tissue samples for gene expression analysis and histopathological assessment were collected simultaneously, it is possible that some transcripts had already been translated into protein and subsequently degraded. Consequently, Hfru−f+ade mice displayed the greatest accumulation of fibrotic protein despite lower mRNA expression levels compared with Ctrl+ade mice.

Consistent with their smaller fibrotic area, Hfru+f10+ade mice exhibited the lowest expression levels of fibrosis-related genes, with significant reductions particularly observed in *Col1a1* and *Acta2*. Collectively, these histological and molecular findings confirm that folate deficiency exacerbates renal fibrogenesis, whereas folate supplementation exerts a protective effect by downregulating profibrotic markers.

### 2.4. Renal Hif1-Alpha and iNos Gene Expression

**Figure 5:**
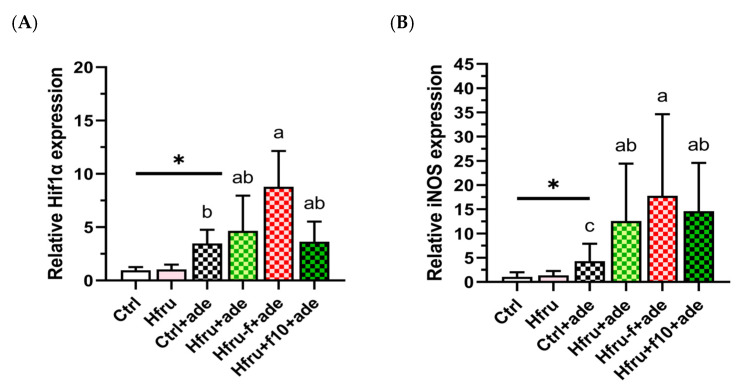
Renal *Hif1α* and *iNOS* gene expression in an adenine-induced murine CKD model under dietary conditions. (**A**) renal *Hif1α* expressions. (**B**) renal *iNOS* expressions. Relative gene expression levels were normalized to *GAPDH*. Values are mean ± SD (n = 12 per group). * *p* < 0.05 versus the Ctrl group. Different letters indicate significant differences among the adenine-induced groups (*p* < 0.05; (**A**) Kruskal–Wallis tests followed by Bonferroni-corrected Mann–Whitney U post hoc tests; (**B**) one-way ANOVA with Duncan’s post-hoc test).

To determine whether long-term high-fructose intake, folate status, and adenine induction contribute to renal hypoxia, renal expression of *Hif1α* and *iNos* was assessed (Figure 5). Renal hypoxia-related gene expression was observed only after adenine induction. As shown in Figure 5A, adenine significantly increased renal *Hif1α* expression, with Hfru−f+ade mice exhibiting higher levels than Hfru+f10+ade mice. A similar pattern was observed for *iNos* expression (Figure 5B), in which folate deficiency enhanced expression, whereas folate supplementation suppressed it. These findings indicate that folate deficiency aggravates renal hypoxia and the associated inflammatory responses in the adenine-induced nephropathy.

### 2.5. Regulatory Cytokine IL-10 in Splenocytes and Renal Tissue

**Figure 6:**
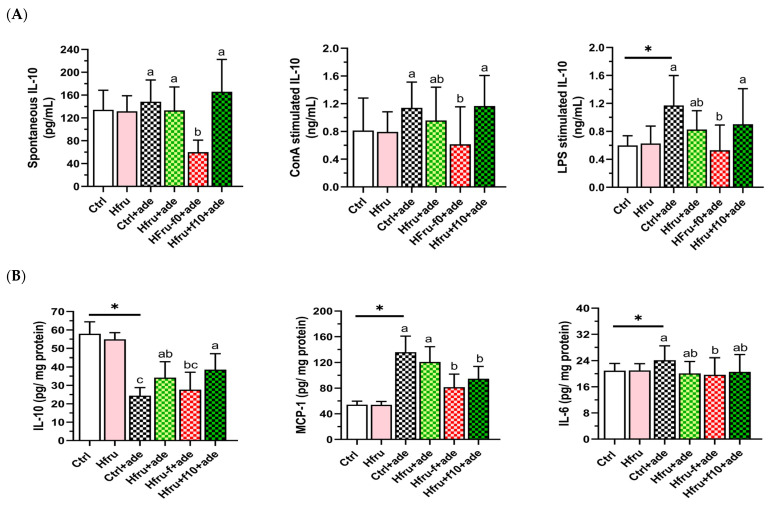
Cytokine secretion and renal cytokine levels in an adenine-induced murine CKD model under dietary conditions. (**A**) cytokine secretion by unstimulated or mitogen-stimulated (ConA or LPS) splenocytes. (**B**) renal cytokine levels. Values are mean ± SD (n = 12 per group). * *p* < 0.05 versus the Ctrl group. Different letters indicate significant differences among adenine-induced groups (*p* < 0.05; one-way ANOVA with Duncan’s post-hoc test).

To assess the effects of high fructose intake, folate status, and adenine induction on immune responses, cytokine secretion by mitogen-stimulated splenocytes and cytokine levels in renal tissue were measured. As shown in Figure 6A, IL-10 secretion by lipopolysaccharide (LPS)-stimulated splenocytes increased significantly following adenine induction. However, Hfru−f+ade mice exhibited significantly lower IL-10 secretion compared with the Ctrl+ade and Hfru+ade groups, whereas IL-10 secretion in Hfru+f10+ade mice did not differ from these groups.

As shown in Figure 6B, renal IL-10 levels were reduced in all adenine-treated groups compared with the Ctrl and Hfru groups. In contrast, pro-inflammatory cytokines, including MCP-1 and IL-6, were significantly elevated following adenine induction. These results indicate that folate status strongly influences IL-10 levels, with a comparatively smaller effect on MCP-1 and IL-6.

Correlations among renal cytokines are presented in Table 1. Renal IL-10 levels were significantly negatively correlated with both MCP-1 and the profibrotic cytokine TGF-β, whereas MCP-1 levels were significantly positively correlated with TGF-β and IL-6. Together, these findings suggest that increased renal IL-10 levels resulting from folate supplementation exert regulatory effects and contribute to the suppression of renal inflammation and fibrosis.

## 3. Discussion

Folate is an essential nutrient and plays a critical role in preventing birth defects. Many countries mandate fortification of staple foods with folic acid to reduce the incidence of neural tube defects. However, diets high in processed foods, frequent medication, and low vegetable consumption are associated with poor nutritional quality [13]. Despite the biological importance of folate, research specifically examining the impact of folate status on CKD progression remains scarce. A 30-year longitudinal study following individuals from young adulthood to midlife reported that lower folate intake in early adulthood was associated with a higher incidence of CKD later in life [14].

We further investigated folate status in combination with a high-fat, high-fructose diet. Our previous study demonstrated that poor folate status in the context of a high-fat, high fructose diet increased lipid accumulation and leptin production in mice fed a diet containing high-fat (45% Kcal from fat, 23.6% *w*/*w*) and high-fructose (31.6% *w*/*w*) for 12 months. However, folate-deficient mice fed a high-fat alone showed smaller increases in serum leptin and hepatic triglyceride levels [12], suggesting high fructose consumption plays a significant role in obesity, as well documented in the literature [14,15,16].

In the present study, the fat content of the experimental diet (25.7% Kcal, 12% *w*/*w*) was reduced to specifically examine the effects of high fructose intake in relation to folate status. Our results showed significantly elevated fasting blood glucose levels after 10 weeks of high-fructose feeding, as well as increased urinary glucose, serum AST activity, serum creatinine, and urinary protein after 17 weeks of feeding. Excess fructose has been reported to disrupt metabolic homeostasis and increase the risk of metabolic disorders, including insulin resistance and fatty liver disease [17]. Our findings further indicate that long-term high fructose intake also impairs the kidney function. However, these indicators were no longer significantly elevated following adenine induction, suggesting that adenine exposure, as an additional stressor, may have masked or overridden the metabolic effects of high fructose in these mice.

CKD was induced in this study by adenine feeding, which leads to 2,8-dihydroxyadenine crystal accumulation, tubular injury, and interstitial fibrosis [18,19]. However, the success of adenine-induced CKD may depend on diet composition. Currently, few studies have examined adenine-induced CKD in mice fed high-fat and high-fructose diets. By adjusting either fat or fructose content in test diets, our preliminary data indicated that diets containing high fat levels, up to 23.6% (*w*/*w*), may hinder adenine absorption and fail to induce nephritis. In addition, mice may exhibit fluctuating appetite during the early phase of high-fructose feeding. Therefore, both diet composition and food intake must be carefully monitored throughout the study.

After 7 weeks of adenine-induction in present study, urine glucose levels were significantly elevated in the adenine-treated groups. However, the effects of high fructose were no longer evident following adenine induction, except in the group receiving high fructose combined with folate deficiency. In contrast, folate supplementation significantly reduced serum creatinine levels and increased creatinine clearance.

Regarding kidney function, adenine significantly induced renal injury markers. Folate deficiency further exacerbated kidney injury, as indicated by increased serum BUN, urinary protein, urinary NGAL, and renal fibrosis. These findings are consistent with our previous study [2]. Notably, renal fibrotic outcomes revealed a discrepancy between molecular fibrotic markers and the severity of histological fibrosis in folate-deficient mice compared with control mice. Although changes in mRNA expression typically precede alterations in protein abundance, protein accumulation may persist even after mRNA expression has declined. This is particularly relevant for fibrotic proteins, which are often long-lived.

The observed discrepancy between histological fibrosis and fibrotic gene expression suggests a temporal dissociation between active fibrogenesis and accumulated extracellular matrix (ECM) deposition. Hfru−f+ade mice may have undergone earlier or prolonged fibrogenic activation, resulting in greater collagen accumulation, even though transcription of fibrotic genes had plateaued or declined after 22 weeks of folate deficiency. Fructose exposure may further suppress steady-state mRNA levels *Col1a1* and *Fn1*, through metabolic and post-transcriptional mechanisms, including altered post-translational regulation, while fibrosis continues to progress at the protein and ECM levels. In contrast, Ctrl+ade mice appear to remain in a more active fibrogenic phase, characterized by higher expression of *Col1a1* and *Fn1* but less ECM accumulation. Conversely, folate supplementation attenuated renal fibrosis, as evidenced by reduced fibrotic area and decreased expression of key fibrotic markers.

With respect to renal cytokines levels and cytokine secretion from activated splenocytes, adenine induction enhanced the response of LPS-stimulated splenocytes, primarily B cells and to a lesser extent macrophage. In the kidney, IL-10 was suppressed, whereas MCP-1 and IL-6 levels were elevated, indicating progression of kidney injury [20]. Long-term folate deficiency tended to reduce cytokine levels, likely due to impaired immune cells proliferation resulting from insufficient folate availability [11,21,22]. In contrast, folate supplementation significantly decreased renal MCP-1 levels and tended to increase IL-10 levels (0.05 < *p* < 0.1). A significant negative correlation was observed between renal IL-10 and the inflammatory cytokine MCP-1, as well as profibrotic cytokine TGF-β, in adenine-induced CKD. Although IL-10 levels were correlated with MCP-1 and TGF-β, these associations do not imply causality and may reflect indirect or parallel regulatory mechanisms. Together, these findings suggest that folate status plays an important role as a reno-protective nutrient. Future mechanistic or intervention studies are warranted to determine whether IL-10 directly modulates these inflammatory and fibrotic pathways.

This study has several limitations. The adenine-induced CKD mouse model and controlled dietary interventions may not fully capture the complexity and heterogeneity of human CKD, and the fructose and folate levels used—including folate deficiency and high-dose supplementation—may not reflect typical human intakes. In addition, species differences in folate metabolism and renal physiology limit direct translational interpretation. Nevertheless, a key strength of this study is the use of a well-controlled dietary design that allowed systematic evaluation of folate status under high-fructose conditions, providing mechanistic insight into renal inflammatory and fibrotic responses. These findings may help inform future studies using more clinically relevant models and human populations.

## 4. Materials and Methods

### 4.1. Animals and Experimental Design

Male C57BL/6JNarl mice (*Mus musculus*), aged 7 weeks with an initial body weight of approximately 25 g, were obtained from the National Laboratory Animal Center (Taipei, Taiwan). All animals were maintained under specific pathogen-free (SPF) conditions and were of wild-type genotype with no prior experimental manipulations. Mice were housed individually to prevent aggression, in a controlled environment (22 ± 2 °C, 50–60% humidity) with a 12-h light/dark cycle, and provided ad libitum access to food and water. Health status and signs of distress (e.g., ruffled fur, lethargy, reduced activity) were monitored daily. Humane endpoints were established as >20% body weight loss from baseline or inability to access food/water; however, no mice reached these endpoints prior to the scheduled sacrifice.

Seventy-two mice were acclimatized to an AIN-93G control diet (Ctrl) for one week and then randomly assigned to six experimental groups (n = 12 per group) at age of 8 weeks, using body-weight stratification to minimize confounding: Ctrl, Hfru, Ctrl+ade, Hfru+ade, Hfru−f+ade, and Hfru+f10+ade. The Ctrl and Ctrl+ade groups received the standard AIN-93G diet, whereas the Hfru groups were fed a high-fructose diet (26% *w*/*w*) (Table A1). The Hfru−f and Hfru+f10 groups received modified high-fructose diets with either folate deficiency (−f) or folate supplementation at tenfold the level of the control diet (+f10), respectively. These diets were administrated for 10 weeks prior to adenine induction. Sample size was determined based on pilot studies to ensure adequate statistical power.

From week 18 to week 25 of age, adenine (1.5 g/kg diet) was added to the diets of the Ctrl+ade, Hfru+ade, Hfru−f+ade, and Hfru+f10+ade groups to induce renal injury. Mice were sacrificed at 25 weeks of age. Body weight and water intake were recorded weekly; food intake was measured every three days. The study was not blinded; investigators were aware of group allocation during all experimental and analytical stages. No animals or data points were excluded from the analysis.

### 4.2. Serum and Urine Collection and Tissue Preparation

Urine was collected by gentle abdominal pressure before adenine feeding and every two weeks thereafter. Blood samples were collected via submandibular vein puncture using a 5-mm lancet. At sacrifice, blood was collected by cardiac puncture after CO_2_ euthanasia. Blood and urine samples were centrifuged at 12,000 rpm for 20 min, and the resulting serum and urine were stored at −80 °C.

Liver and kidney tissues were homogenized individually in RIPA buffer containing 1% protease inhibitor using bead-based mechanical disruption. Homogenates were centrifuged at 20,000× *g* for 10 min at 4 °C, and the supernatants were collected and stored at −20 °C for further analysis, including protein quantification, renal cytokine levels, and hepatic folate content.

Splenocytes were isolated from freshly excised spleens by mechanical dissociation through a cell strainer, followed by red blood cell lysis and washing with RPMI-1640 medium. Splenocytes were cultured in complete RPMI and stimulated with LPS (100 ng/mL), ConA (20 ng/mL), or combined treatment at 37 °C in 5% CO_2_. After incubation, culture supernatants were collected for cytokine determination.

### 4.3. Measurements of Folate, Glucose and Liver Enzymes

Serum and hepatic folate levels were determined using a microbiological assay based on the folate-dependent growth of *Lactobacillus casei* (ATCC7469). Serum samples or folate standards were added to 96-well plates containing folate-free casei medium inoculated with *L. casei* and incubated at 37 °C for 18–22 h. Bacterial growth was measured at 620 nm, and folate concentrations were calculated from a standard curve [11].

Serum and urinary glucose concentrations were measured using a glucose GOD-PAP colorimetric assay (Randox Laboratories, Crumlin, UK). Absorbance was read at 520 nm after 10 min of incubation at 37 °C with the glucose reagent, and glucose levels were calculated relative to the standard curve.

Serum alanine aminotransferase (ALT) and aspartate aminotransferase (AST) activities were measured using Randox colorimetric kits (AL1268, AS1204). Absorbance at 340 nm was recorded at 1-min intervals, and enzyme activities were calculated as 1746 × ΔOD_340_/min.

### 4.4. Determination Renal Function

#### 4.4.1. Serum Biomarkers

Serum blood urea nitrogen (BUN) and creatinine were measured using the QuantiChrom™ Urea Assay Kit (DIUR-100; BioAssay Systems, Hayward, CA, USA) and QuantiChrom™ Creatinine Assay Kit (DICT-500; BioAssay Systems, Hayward, CA, USA), respectively, following the manufacturer’s colorimetric protocols. Absorbance was recorded at 520 nm, and concentrations were derived from standard curves.

#### 4.4.2. Urine Biomarkers

Urinary creatinine was quantified using the QuantiChrom™ Creatinine Assay Kit (DICT-500; BioAssay Systems, Hayward, CA, USA). Urinary total protein was determined using the Pierce™ Coomassie (Bradford) Protein Assay Kit (Thermo Fisher Scientific, Waltham, MA, USA) and normalized to urinary creatinine. Creatinine clearance (Ccr) was calculated using the formula: Ccr (mL/min) = (Ucr × V)/(Scr × 1440), where Ucr is the urinary creatinine concentration, V is the 24-h urine volume, Scr is the serum creatinine concentration, and 1440 represents the number of minutes in 24 h.

Urinary NGAL levels were measured using the Mouse NGAL ELISA Kit (R&D Systems, Minneapolis, MN, USA), and urinary KIM-1 levels were determined using the Mouse KIM-1 ELISA Kit (Innovative Gene Technologies, San Diego, CA, USA). Absorbance was measured at 450 nm, and concentrations were calculated using standard curves.

#### 4.4.3. Renal Histopathological Analysis

Half-kidneys were fixed in 10% formalin, paraffin-embedded, sectioned, and stained with hematoxylin and eosin (H&E) or Masson’s trichrome. Immune cell infiltration and renal injury were evaluated, and fibrosis was quantified using Fiji (an open-source distribution of ImageJ v1.54f; National Institutes of Health, Bethesda, MD, USA).

### 4.5. Renal mRNA Expression Analysis

#### 4.5.1. RNA Extraction

Total RNA was extracted from kidney tissue using TRIzol reagent, followed by chloroform extraction, isopropanol precipitation, ethanol washing, and resuspension in RNase-free water.

#### 4.5.2. cDNA Synthesis and qPCR

cDNA was synthesized from 200 ng of total RNA using the High-Capacity cDNA Reverse Transcription Kit. Quantitative PCR was performed using SYBR Green Supermix on a Bio-Rad CFX system. mRNA expression of *Hif1α*, *iNOS*, *Col1a1*, *Fn1*, and *Acta2* was quantified, with *GAPDH* as the internal reference gene. Primer sequences and qPCR cycling conditions are listed in Table 2. Relative gene expression was calculated using the 2^−ΔΔCt^ method.

### 4.6. Cytokine Analysis

Cytokines, including IL-6, IL-10, MCP-1, and TGF-β from cultured splenocyte or kidney homogenate were quantified using ELISA kits from BioLegend (San Diego, CA, USA) and Invitrogen (Carlsbad, CA, USA). ELISA procedures were performed according to the manufacturer’s protocols, including plates coating (when applicable), incubation with detection antibodies, HRP-conjugate binding, and substrate development. Absorbance was measured at 450 nm, and cytokine concentrations were calculated from standard curves.

### 4.7. Statistical Analysis

All continuous variables were tested for normality (Shapiro–Wilk) and homogeneity of variance (Levene’s test) (Supplementary Table S1A). Student’s *t*-test was used for comparison between two groups, and one-way ANOVA followed by Duncan’s post hoc test was applied for multiple-group comparisons. When variables violated, Kruskal–Wallis test followed by Mann–Whitney U tests with Bonferroni correction was used. Data are presented as mean ± SD, with additional descriptive statistics (median, IQR) shown in Supplementary Table S1B. Statistical significance was defined as an adjusted *p* < 0.05. Group differences are indicated by different letters (a, b, ab, etc.), with shared letters denoting no significant difference. Statistical analyses were performed using SPSS version 24.0 (IBM Corp., Armonk, NY, USA), and figures were generated using GraphPad Prism version 9.0 (GraphPad Software, San Diego, CA, USA).

## 5. Conclusions

To investigate whether dietary factors influence the progression of chronic kidney disease (CKD), this study modeled a modern dietary pattern characterized by high intake of sugar/fructose syrup and reduced consumption of folate-rich vegetables. Mice fed a high-fructose diet developed obesity and exhibited higher blood and urinary glucose levels, increased serum aspartate aminotransferase (AST) activity and serum creatinine, and elevated urinary protein prior to adenine induction. In adenine-induced CKD mice, folate deficiency significantly increased blood urea nitrogen (BUN), urinary protein, and neutrophil gelatinase-associated lipocalin (NGAL), whereas folate supplementation significantly reduced serum creatinine, and increased creatinine clearance. Overall, our study demonstrated that both fructose intake and folate status influence CKD progression through modulation of renal inflammation and fibrosis, highlighting the importance of adequate folate intake in maintaining kidney health.

## Figures and Tables

**Table 1:** Pearson correlation analysis among renal cytokine levels in an adenine-induced murine model of chronic kidney disease.

Kidney		IL-10	IL-6	TGF-β
MCP-1	r value	−0.656	0.293	0.645
	*p* value	<0.0001	0.013	<0.0001
TGF-β	r value	−0.612	0.115	
	*p* value	<0.0001	0.359	
IL-6	r value	−0.061		
	*p* value	0.616		

Correlation coefficients (r values) were calculated using Pearson’s correlation analysis (n = 71, *p* < 0.05).

**Table 2:** Primers used in this study.

Gene Name	Forward Primer (5′ to 3′)
*Collagen I*	F: TTC AGC TTT GTG GAC CTC CG R: TTG CAC GTC ATC GCA CAC AG
*Fibronectin*	F: GCA GTG ACC ACC ATT CCT G R: GGT AGC CAG TGA GCT GAA CAC
*Collagen IV*	F: TCC TTG TGA CCA GGC ATA GT R: TTG AAC ATC TCG CTC CTC TC
*Hif1α*	F: CTA TGG AGG CCA GAA GAG GGT AT R: CCC ACA TCA GGT GGC TCA TAA
*iNOS*	F: TCC AAG GTA TCC TGG AGC GA R: CAG GGA CGG GAA CTC CTC TA
*GAPDH*	F: GGT GAA GGT CGG TGT GAA CG R: CTC GCT CCT GGA AGA TGG TG

## Data Availability

All data generated or analyzed during this study are included in the article and Supplementary Materials. Further inquiries can be directed to the corresponding author.

## Supplementary Materials

The following supporting information can be downloaded at: https://www.mdpi.com/article/10.3390/ijms27010499/s1.

## Abbreviations

The following abbreviations are used in this manuscript:

Ade	Adenine
ALT	Alanine aminotransferase
AST	Aspartate aminotransferase
BUN	Blood urea nitrogen
CKD	Chronic kidney disease
ConA	Concanavalin A
ELISA	Enzyme-linked immunosorbent assay
GABA	Gamma-aminobutyric acid
*GAPDH*	Glyceraldehyde 3-phosphate dehydrogenase
H&E	Hematoxylin and eosin
Hfru	High-fructose diet
Hif1α	Hypoxia-inducible factor 1 alpha
*iNOS*	Inducible nitric oxide synthase
IL-6, 10, 17A	Interleukin-6, 10, 17A
KIM-1	Kidney injury molecule-1
LPS	Lipopolysaccharide
MCP-1	Monocyte chemoattractant protein
NGAL	Neutrophil gelatinase-associated lipocalin
qPCR	Quantitative polymerase chain reaction
TGF-β	Transforming growth factor beta
TNF-α	Tumor necrosis factor alpha
α-SMA	Alpha-smooth muscle actin

## Appendix A

**Table A1:** AIN-93G purified diet composition ^1^.

Ingredient ^2^ (g/kg)	Ctrl ^3^	Hfru ^3^	Hfru−f ^3^	Hfru+f10 ^3^
Folic acid (mg)		2	0.2	20
Lard		110	110	110
Soybean oil	70	10	10	10
Fructose		264	264	264
Sucrose	100	100	100	100
Corn starch	529.5	206	206	206
Casein	200	206	206	206
Cellulose	50	51	51	51
AIN-93G Mineral mix	35	37	37	37
AIN-93G Vitamin mix (W/O folic acid)	10	10.5	10.5	10.5
L-Cystine	3	3	3	3
Choline	2.5	2.5	2.5	2.5
Lard		110	110	110
Total energy (kcal)	3960	4196	4196	4196
Fat (% kcal)	15.9	25.7	25.7	25.7
CHO (% kcal)	63.6	54.3	54.3	54.3
Protein (% kcal)	20.5	19.9	19.9	19.9

^1^ Reeves et al., 1997 [23] ^2^ folic acid, casein, and choline from Sigma-Aldrich (St. Louis, MO, USA); Lard, AIN-93G Mineral mix, AIN-93G Vitamin mix (MP Biomedicals, Irvine, CA, USA); Soybean oil, Sucrose (Taiwan Sugar Corp., Tainan, Taiwan); Fructose (ADM^®^, Chicago, IL, USA); Corn starch (Samyang, Seoul, South Korea); Cellulose (JRS PHARMA, Rosenberg, Germany); L-Cystine (Wako Pure Chemical Industries, Osaka, Japan) ^3^ The diets for the adenine groups were supplemented with 0.1–0.2% (1–2 g/kg) pure adenine (Sigma-Aldrich).

**Table A2:** Caloric intake and adenine consumption before and after adenine diet ^1^.

Group	Before Adenine	After Adenine	Total	Adenine Intake
	Relevant Energy Intake (kcal/BW (g))			(mg/BW (g))
Ctrl	0.46 ± 0.08	0.38 ± 0.10	0.42 ± 0.09	N/A
Hfru	0.48 ± 0.05	0.36 ± 0.09	0.41 ± 0.08	N/A
Ctrl+ade	0.44 ± 0.02	0.37 ± 0.04	0.38 ± 0.03	0.12 ± 0.02
Hfru+ade	0.49 ± 0.04	0.35 ± 0.06	0.40 ± 0.05	0.10 ± 0.02
Hfru−f+ade	0.48 ± 0.07	0.35 ± 0.07	0.38 ± 0.07	0.11 ± 0.04
Hfru+f10+ade	0.47 ± 0.06	0.33 ± 0.04	0.38 ± 0.04	0.10 ± 0.01

^1^ Relative energy intake and adenine intake divided by body weight is expressed as calories per gram (kcal/g) and mg per gram (mg/g), respectively. Values were mean ± SD. n = 12/group. There was no significant difference after one-way ANOVA followed by Duncan’s post hoc test.
