# Supplementary material for: Effects of Folate and Fructose Intakes on Renal Cytokines and Fibrosis in an Adenine-Induced Mouse Model of Chronic Kidney Disease
Source: Int J Mol Sci. 2026 Jan 3;27(1):499. doi: 10.3390/ijms27010499 (PMC12787204; doi:10.3390/ijms27010499)
Supplement: Supplementary file 1 [file ijms-27-00499-s001.zip › ijms-4046815-supplementary.pdf]

Supplementary Table

S1. Assessment of statistical assumptions and descriptive statistics for continuous variables.

(A) Tests of normality and homogeneity of variance for each variable.

Group	Shapiro–Wilk W	p value	Levene's F	p value	Parametric
Serum ALT	0.769	0.006 *	5.778	0.002 *	No
Urine Glucose	0.833	0.085	5.257	0.006 *	No
Urinary NGAL	0.869	0.222	8.760	<0.001*	No
Relative HIF1α	0.767	0.029 *	2.511	0.086	No

Data were assessed for normality using the Shapiro–Wilk test and for homogeneity of variance using Levene's test. Parametric suitability was defined as Shapiro–Wilk p ≥ 0.05 and Levene' s p ≥ 0.05.

(B) Descriptive statistics (median, IQR, mean ± SD) for each +ade group.

Variable	Group	n	Median	IQR	Mean ± SD
ALT	Ctrl+ade	12	27.9	22.2-36.3	29.3 ± 3.2
	Hfru+ade	12	22.7	20.0-27.7	23.8 ± 17
	Hfru-**f**+ade	12	47.1	34.7-68.6	51.7 ± 7.3
	Hfru+**f10**+ade	12	18.8	17.6-23.7	20.6 ± 1.3
Urine Glucose	Ctrl+ade	12	49.07	391.9-544.2	468.0 ± 31.1
	Hfru+ade	12	214.9	159.6-291.3	225.5 ± 25.6
	Hfru-**f**+ade	12	687.4	449.8-834.4	642.1 ± 69.3
	Hfru+**f10**+ade	12	252.4	153.9-474.9	314.4 ± 57.8
Urinary NGAL	Ctrl+ade	12	12.5	9.9-13.8	11.8 ± 0.8
	Hfru+ade	12	5.3	7.3-3.8	5.6 ± 0.6
	Hfru-**f**+ade	12	17.6	11.4-21.4	16.4 ± 1.8
	Hfru+**f10**+ade	12	6.3	3.2-11.1	7.2 ± 1.4
Relative HIF1α	Ctrl+ade	12	3.2	2.3-4.6	3.5 ± 0.5
	Hfru+ade	12	3.1	1.1-8.1	4.6 ± 1.3
	Hfru-**f**+ade	12	10.6	6.5-13.0	9.8 ± 1.2
	Hfru+**f10**+ade	12	3.7	2.1-6.2	4.1 ± 0.7

Data were assessed for normality using the Shapiro–Wilk test and for homogeneity of variance using Levene' s test. Parametric suitability was defined as Shapiro–Wilk p ≥ 0.05 and Levene's p ≥ 0.05.
